# Case Report: Giant Congenital Left Atrial Appendage Aneurysm Presenting With Acute Massive Cerebral Infarction and Refractory Atrial Fibrillation: A Case Report and Literature Review

**DOI:** 10.3389/fcvm.2022.888825

**Published:** 2022-05-10

**Authors:** Rui Li, Fei Ma, Han Xiong Guan, Yue Ying Pan, Li Gang Liu, Dao Wen Wang, Hong Wang

**Affiliations:** ^1^Division of Cardiology, Department of Internal Medicine, Hubei Key Laboratory of Genetics and Molecular Mechanism of Cardiologic Disorders, Tongji Hospital, Tongji Medical College, Huazhong University of Science and Technology, Wuhan, China; ^2^Department of Radiology, Tongji Hospital, Tongji Medical College, Huazhong University of Science and Technology, Wuhan, China; ^3^Division of Cardiothoracic and Vascular Surgery, Tongji Hospital, Tongji Medical College, Huazhong University of Science and Technology, Wuhan, China

**Keywords:** left atrial appendage aneurysm, echocardiography, atrial fibrillation, acute cerebral infarction, case report

## Abstract

**Background:**

Congenital left atrial appendage aneurysm (LAAA) is a rare cardiac anomaly with a variety of presentations, from being asymptomatic to potentially serious complications such as systemic thromboembolism and atrial tachyarrhythmia.

**Case Presentation:**

We report a case of congenital giant LAAA in a 35-year-old man presenting with acute massive cerebral infarction and atrial fibrillation (AF) with rapid ventricular rate. The AF was refractory to conventional antiarrhythmic agents, such as amiodarone and electrical cardioversion, but restored and maintained sinus rhythm after surgical resection of LAAA. The patient remained free of events and was in sinus rhythm during half-year follow-up.

**Conclusion:**

Giant LAAA has the potential causing serious complications and should be managed surgically in most cases.

## Introduction

Left atrial appendage aneurysm (LAAA) is a rare entity that can be congenital or acquired in etiology (1, 2). Congenital LAAAs are caused by dysplasia of pectinate muscles in the appendage (3). A lot of LAAAs were asymptomatic and were discovered incidentally during echocardiographic exams (4, 5). Others developed symptoms or signs after the second to third decade of life, such as palpitations, chest pain, dyspnea on exertion, and atrial tachyarrhythmia (6–9). A small percentage of patients were diagnosed after complications, mainly systemic thromboembolism (10–14). Surgical resection is the recommended standard therapy in the literature although some reports suggested the conservative management with clinical monitoring as an optional strategy in some asymptomatic patients (13, 15, 16). Herein, we present a case of a giant LAAA in a 35-year-old man who presented with acute massive cerebral infarction and refractory atrial fibrillation (AF) with rapid heart rate. Of interest, sinus rhythm was restored after LAAA resection.

## Case Presentation

**FIGURE 1:**
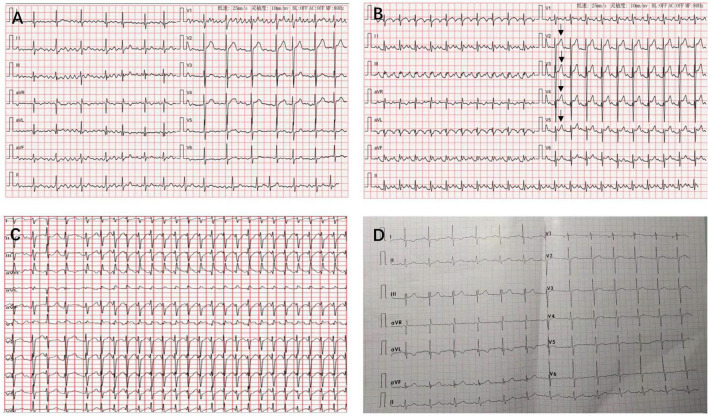
Twelve-lead electrocardiograms revealing atrial fibrillation **(A)**, atrial flutter with mild ST-segment elevation in leads V2–V5 (arrows) **(B)**, atrial tachycardia with a rapid ventricular rate of 238 beats/min was recorded when he was experiencing attacks of atrial tachycardia **(C)**, and sinus rhythm at 6-month follow-up **(D)**.

**FIGURE 2:**
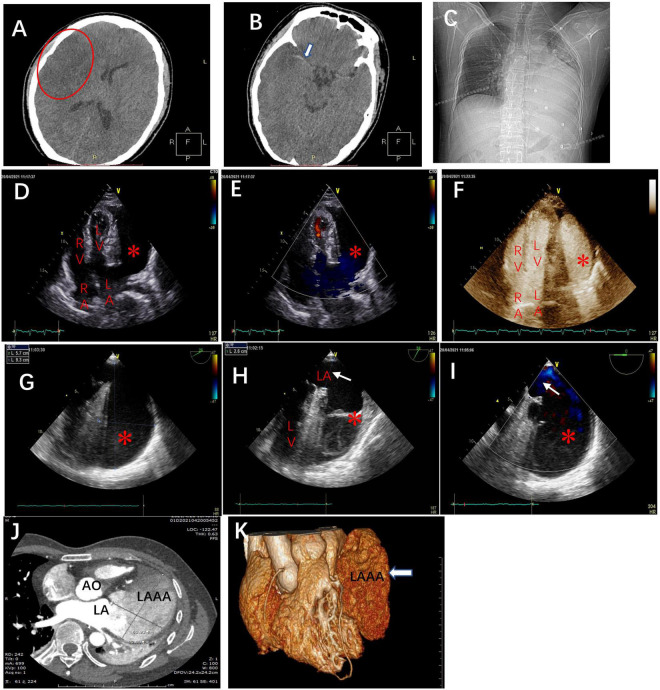
The brain computed tomography scans at admission revealed **(A)** massive cerebral infarction in the right-sided frontotemporal insula (red circle) and **(B)** the basal ganglia area with right middle cerebral artery thrombus (white arrow). **(C)** The chest X-ray in the cardiology department demonstrating the abnormal left heart border. Transthoracic echocardiography (TTE), apical four-chamber view **(D)**, and the view with color doppler **(E)** revealing an echo-free cavity (red star) adjacent to and compressing the LV and the probable communication of the cavity with the LA; TTE with contrast showing the contrast filling in the LA and the cavity simultaneously without filling defect in the cavity **(F)**; TEE, the mid-esophageal 2-chamber and LAA view demonstrating a giant cavity (red star) measuring 9.3 cm × 5.7 cm **(G)** and its connection to LA *via* a 2.6 cm-wide orifice (white arrow) **(H)** and the to-and-fro blood flows between LA and the cavity (red star) through the orifice (white arrow) **(I)**. Axial computed tomography scan **(J)** and 3D reconstruction **(K)** demonstrate a 9.3 cm × 6.4 cm × 3.8 cm giant LAAA. AO: aorta; RA: right atrium; LA: left atrium; RV: right ventricle; LV: left ventricle.

A 35-year-old man who had no previous medical history and cardiac history suddenly lost consciousness and was referred to our hospital 10 h after the onset of the stroke. At the time of admission, his coma was rated 7 on the Glasgow Coma Scale (GCS), and he displayed left-sided hemiplegia. He had sinus rhythm with paroxysmal AF (Figure 1A) and normal blood pressure (111/68 mmHg). Computed tomography (CT) revealed massive cerebral infarction in the right-sided frontotemporal insula (Figure 2A) and the basal ganglia area with the right middle cerebral artery thrombus (Figure 2B). Due to the massive infarction, he was referred to a neurosurgeon who performed decompressive craniectomy (DC) plus temporalis muscle attachment treatment at 26 h after admission. The patient’s mental status improved and became clear 10 days postoperatively, although he was still having left hemiplegia and aphasia. Thereafter, he was experiencing paroxysmal atrial tachycardia with mild ST-segment elevation in leads V2–V5 (Figure 1B). On day 16, the patient was then referred to the cardiology department for further cardiac evaluation.

The chest x-ray revealed a greatly enlarged left border of the heart (Figure 2C). Transthoracic echocardiography (TTE) (Vivid E9; GE Healthcare, Norway) revealed a giant echo-free cystic structure adjacent to the posterolateral wall of the left ventricle (LV) and compressed the anterolateral LV wall during the entire cardiac cycle (Figure 2D). The apical 4-chamber views with color Doppler demonstrated that this cavity was probably related to and communicated with the left atrium (LA) *via* a broad neck (Figure 2E). Contrast-enhanced echocardiography was performed then, which showed that the contrast agent filled the LA and the cystic structure was simultaneously with no filling defect (Figure 2F). Further transesophageal echocardiography (TEE) confirmed that the enlarged cavity measuring 9.3 cm × 5.7 cm was arising from the LA and communicating with the LA through a 2.6 cm-wide orifice, with a big chicken-wing morphology and with intense spontaneous echo contrast but no obvious thrombi (Figures 2G,H). To-and-fro blood flows through the orifice were detected by color Doppler image (Figure 2I). There were no other cardiac anomalies, and congenital LAAA was diagnosed. Computed tomography (CT) confirmed the echocardiographic findings and showed a giant LAAA measuring 9.3 cm × 6.4 cm × 3.8 cm that was compressing the LV wall (Figures 2J,K).

On day 17, the patient was frequently experiencing attacks of atrial tachycardia with a very rapid ventricular rate of 200–240 beats/min (Figure 1C) and reduced blood pressure to 80–90/50–60 mmHg. The AF was refractory to conventional antiarrhythmic agents such as amiodarone. Standard biphasic electrical cardioversion was performed several times, which could not convert AF but only maintain sinus rhythm for a short time. Intravenous injections of beta-blocker continuously and cedilanid intermittently were applied to slow the heart rate. Given the potential risk of thromboembolic events and the refractory AF with rapid heart rate causing hemodynamic instability, prompt surgical resection of LAAA was considered. Due to the high bleeding risk of the patient, anticoagulation therapy was not performed by oral anticoagulants but by using low molecular weight heparin, after the electrical cardioversion.

**FIGURE 3:**
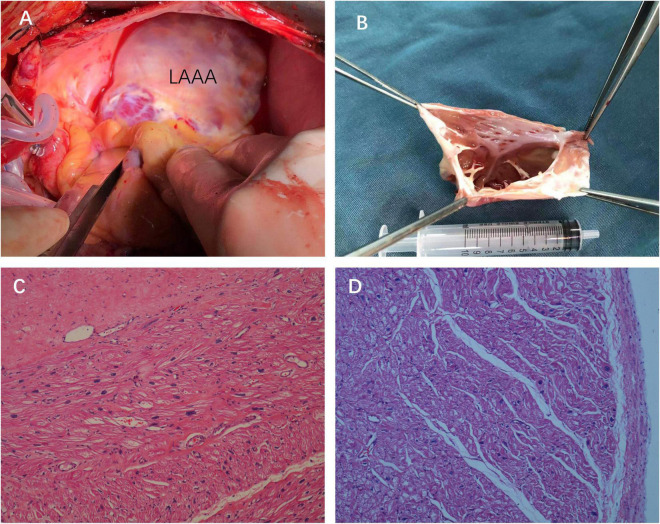
Intraoperative views **(A)** and the surgical specimen of the LAAA with no thrombus inside the cavity of the aneurysm **(B)**. Hematoxylin and eosin staining of the resected left atrial appendage tissues showing the wall of the aneurysm composed of the myocardium and fibrotic tissue [**(C,D)**, 2007×].

The patient was then referred to the cardiothoracic surgery department the next day. He underwent LAAA resection through median sternotomy aided by cardiopulmonary bypass on day 37. A huge LAAA measuring about 9.5 cm in long axis was visualized with intact pericardium during the operation (Figures 3A,B). There was no thrombus inside the cavity of the aneurysm (Figure 3B). Histopathology examination revealed focal chronic inflammation with myocardial atrophy and degeneration and increased interstitial fibrosis, which are typical histological features of LAAA (Figures 3C,D).

The patient had an uneventful postoperative course. Interestingly, the sinus rhythm was restored and maintained since LAAA resection. He was discharged home in sinus rhythm 6 days post LAAA resection. Antiarrhythmic agents were not used after resection, and the anticoagulation agents were discontinued 1 month later after discharge. At 6-month follow-up, he was still in sinus rhythm (Figure 1D) and free of adverse events. Table 1 summarizes the clinical presentation and management of the patient.

**TABLE 1:** Timeline of events.

Days (d)	● Events
D1	● Lost consciousness and referred to our hospital 10 h later, left-sided hemiplegia.
	● GLS 7, AF, normal blood pressure
	● Head CT revealed massive cerebral infarction and right middle cerebral artery thrombus referred to neurosurgery
D2	● Decompressive craniectomy (DC)
D16	● AF, transferred to cardiology
	● Chest x-ray, TTE, TEE, Contrast-enhanced echocardiography, Coronary CT angiography clarified the diagnosis of left atrial appendage aneurysm
D17	● Frequently attacks of atrial tachycardia, refractory to conventional antiarrhythmic agents and electrical cardioversion
D18	● Transferred to cardiothoracic surgical ward
D37	● LAAA resection
D43	● Discharged from hospital

## Discussion

Left atrial appendage aneurysm is a rare abnormality characterized by either local or diffuse outpouching and enlargement of the left atrial appendage (17), which is generally considered to be either congenital or acquired as a result of mitral valve disease or syphilitic myocarditis (18). Herein, we report on a rare congenital giant LAAA, causing acute cerebral infection and refractory AF. Due to the unusual initial presentation of the patient, prompt diagnosis and proper management were challenging and required interdisciplinary considerations.

Congenital LAAA was firstly described in two children by Dr. Parmley in 1962 (19). Most studies about LAAA are individual case reports (4, 8, 13), and to date, about 150 cases of this defect have been reported in the literature (7, 10, 20). Although this disease is rare, its consequences are potentially hazardous and late diagnosis is common. In addition, no widely accepted consensus exists in regard to the management of this entity.

The presentation of LAAA was greatly variable. About one-third of LAAA was discovered incidentally during the echocardiographic exam and was asymptomatic at the time of diagnosis (21). Others develop symptoms or signs of the disease in about the third decade of life (13). Some symptoms of this disease may not be related to the heart, so that the diagnosis is challenging in such cases. The most common manifestations are heart palpitations (43%), shortness of breath (22%), heart rhythm disturbances (15%), embolic disorders of cerebral circulation (11%), and chest pain, and discomfort (7%) (8, 22). Cough and hiccups have been described as very rare and atypical presentations (7, 8). Heart rhythm disturbances, mainly supraventricular tachycardia and AF, occur as a result of structural remodeling of the LAA (9, 22). Finally, thromboembolic events may occur as a serious complication of AF and LAA dilatation with subsequent blood stasis and thrombosis of the aneurysmal cavity (10, 23). In the literature, there are a small number of LAAA being diagnosed after thromboembolic events, such as stroke (24).

In our case, the patient presented with cerebral infarction, a thromboembolic complication of LAAA. His cerebral infarction was massive and required decompressive craniectomy, which has not been reported before. Due to the initial neurological presentation, the diagnosis of LAAA in the case was delayed until the patient suffered AF episodes. Moreover, the AF in the patient was with extremely quick heart rate that was refractory to antiarrhythmic agents and electrical cardioversion and causing dropping of blood pressure. Interestingly, during the AF episode, the ST-segment elevation in the precordial leads was recorded. As previously reported, it was likely caused by embolic occlusion of a coronary artery (11) or by external compression of the coronary arteries by LAAA (20, 25, 26). In our case, the compression of the left coronary artery or its divisions was more likely since no elevation of serial myocardial injury biomarkers was recorded.

The following diagnostic criteria for this congenital form have been proposed by Foale et al.: (1) origin from an otherwise normal atrial chamber; (2) well-defined communication with the atrial cavity; (3) location within the pericardium; and (4) distortion of the LV by an aneurysm (10, 27). There are two types of LAAA, extrapericardial and intrapericardial types (18). In extrapericardial type, the LA or LAA is prolated through the pericardial defect and compressed in it, which leads to the aneurysmal expansion of the extrapericardial part of LA. The case we presented and discussed here was intra pericardial type of LAAA.

According to the above criteria, imaging exams are essential tools for diagnosis and precise evaluation of LAAA, which are important for the subsequent treatment (28). Imaging modalities used to diagnose the LAAA include chest x-ray, echocardiography, CT, and magnetic resonance imaging (MRI) (10, 18). Convexity of the LA contour on a chest x-ray should raise the possibility of LAAA and require a differential diagnosis from a pericardial cyst and heart or mediastinal tumors (15, 18). TTE is considered the primary method of diagnosis but with low diagnostic accuracy and sensitivity. TTE usually shows a large saccular echo-free structure laterally to LV. It can, however, hardly in most cases show the connection and communication between the LA and the echo-free cavity and, therefore, cannot make the definite diagnosis of LAAA in many cases and sometimes make the diagnosis mistakenly as a pericardial cyst or effusion (3, 17). Khaled A. Shams reported a case with aneurysmally dilated LA pushing the heart to the right side, and thus a dextro-posed heart was misdiagnosed as dextro-cardia with rheumatic heart disease and AF for a long time till the disease progressed to cardiogenic shock (29). Contrast-enhanced echocardiography can demonstrate the connection of LA and the cavity, which clearly shows the LAAA border, and more importantly the presence of a thrombus, which is a key element for therapeutic choice (21). TEE can provide clear visualization of the structure and composition of the LAAA and its connection with LA and should be mandatory if the diagnosis is ambiguous after TTE (30). Cardiac CT and MRI can clearly visualize LAAA with similar diagnostic accuracy as TEE. Moreover, they are more advantageous in assessing surrounding structures as probable compression of left coronary arteries and pulmonary veins. In most cases and in our case, LAAA was initially diagnosed by contrast-enhanced echocardiography and TEE and was further confirmed by cardiac CT or MRI (18, 21).

The early surgical intervention seems to be the standard of treatment once the diagnosis is established in the current literature, even in asymptomatic cases, aimed to prevent the occurrence of serious complications (10, 31). However, some reports suggested conservative management without or with delayed surgical treatment, which may be an option for patients with no or mild symptoms (13, 15). Because of the rarity of this entity, a direct comparison of medical treatments such as anticoagulation and antiarrhythmic therapies with surgical intervention is not available in the current literature. Thus, if conservative management was chosen, the patient should be closely monitored and anticoagulants may be considered (13, 15).

The recommendation of surgical occlusion in symptomatic patients is based on non-randomized or observational cohort studies (32) as it can prevent thromboembolic complications and atrial arrhythmias associated with the LA enlargement. Transcatheter device occlusion (33) has also been reported for the occlusion of narrow neck LAAs with a high rate of success (34). Available data in small studies suggest that LAA closure using LARIAT epicardial suture is a good alternative for stroke risk reduction (35). However, the incidence of major complications such as perforation of the left atrial appendage, pleural effusion, and thrombosis is also high, and more data regarding device safety and efficacy are needed (36). Moreover, due to the size limitations of currently available occluders, usually not larger than 33 mm, there is no report of device occlusion in really giant LAA by far (37).

Various successful approaches to aneurysmectomy with or without cardiopulmonary bypass have been described, such as median sternotomy, left thoracotomy, mini-thoracotomy, and endoscopy (1, 38). Recently, catheter closure of a giant LAAA was reported, providing a novel non-invasive approach to treating LAAA (3). Removal of the aneurysm usually results in complete resolution of atrial arrhythmias as seen in previous reports (20, 39), although a concomitant full maze procedure was suggested in another study of LAAA with chronic AF. However, concomitant AF surgery may increase the risk of requiring a permanent pacemaker (40). In our case, considering the large size of LAAA, resection *via* median sternotomy and cardiopulmonary bypass was used. A full maze procedure was not adopted in the patient, but sinus rhythm was restored right after the operation and maintained as of the time of writing.

In our opinion, surgical intervention should be taken in symptomatic LAAA or in secondary prevention of thromboembolic events. In asymptomatic cases or in primary prevention, risk stratification for thromboembolic events is the key for the choice of management strategies. The early surgical intervention should be taken in cases with a high risk of thromboembolic events. However, in addition to spontaneous echo contrast and thrombi in the aneurysm, no other reliable indicators can predict embolic complications (21). A study suggested that the presence of AF/flutter was the only predictor of thrombus formation and embolic events (10). Whether the size, orifice, and morphology of LAAA can predict embolic events need to be studied in the future.

## Limitations

This is only a case report with a literature review, and such a study has inherent limitations and may not be able to provide the true perspective of the disease. Again, the LAAA is a very rare disease and a case series study is hard to perform in the real world. Also, this study does not allow us to establish a cause–effect relationship between LAAA and massive cerebral infarction.

## Conclusion

We described here a unique case with a giant LAAA that presented with acute massive cerebral infarction and refractory AF with very rapid heart rate, which has never been described before in the literature. LAAA was detected initially by TTE and thereafter confirmed by contrast-enhanced echocardiography and TEE. Surgical resection was performed promptly after frequently occurring AF with rapid heart rate, and the patient had favorable outcome. Giant LAAA has a high risk of causing significant morbidity as in our case and should be managed by surgical intervention in most cases.

## Data Availability Statement

The original contributions presented in the study are included in the article/supplementary material, further inquiries can be directed to the corresponding author.

## Ethics Statement

Written informed consent was obtained from the individual(s) for the publication of any potentially identifiable images or data included in this article.

## Author Contributions

RL wrote the report, performed the literature research, and took the pictures. RL and FM performed echocardiography, including TEE and contrast-enhanced echocardiography. LL performed surgery. HG and YP provided CT images. HW revised the report and performed the literature research. DW reviewed the manuscript. All authors read and approved the final manuscript.

## Conflict of Interest

The authors declare that the research was conducted in the absence of any commercial or financial relationships that could be construed as a potential conflict of interest.

## Publisher’s Note

All claims expressed in this article are solely those of the authors and do not necessarily represent those of their affiliated organizations, or those of the publisher, the editors and the reviewers. Any product that may be evaluated in this article, or claim that may be made by its manufacturer, is not guaranteed or endorsed by the publisher.

## Abbreviations

LAA: left atrial appendage
LAAA: left atrial appendage aneurysm
TTE: transthoracic echocardiography
TEE: transesophageal echocardiography
CEUS: contrast-enhanced ultrasound
CT: computed tomography
MRI: magnetic resonance imaging
AF: atrial fibrillation
AO: aorta
LA: left atrium
LV: left ventricle
RA: right atrium
RV: right ventricle
SVT: supraventricular tachycardia
ECG: electrocardiogram.
